# Dexamethasone-Induced Perturbations in Tissue Metabolomics Revealed by Chemical Isotope Labeling LC-MS Analysis

**DOI:** 10.3390/metabo10020042

**Published:** 2020-01-21

**Authors:** Lina A. Dahabiyeh, Abeer K. Malkawi, Xiaohang Wang, Dilek Colak, Ahmed H. Mujamammi, Essa M. Sabi, Liang Li, Majed Dasouki, Anas M. Abdel Rahman

**Affiliations:** 1 Division of Pharmaceutical Sciences, School of Pharmacy, The University of Jordan, Amman 11942, Jordan; L.Dahabiyeh@ju.edu.jo; 2 Department of Chemistry and Biochemistry, Concordia University, 7141 Sherbrook Street West, Montréal, QC H4B 1R6, Canada; abeer.malkawi@mail.concordia.ca; 3 Department of Comparative Medicine, King Faisal Specialist Hospital and Research Center (KFSHRC), Riyadh 11461, Saudi Arabia; 4 Department of Chemistry, University of Alberta, Edmonton, AB T6G 2G2, Canada; xi14@ualberta.ca (X.W.); liang.li@ualberta.ca (L.L.); 5 Department of Biostatistics, Epidemiology and Scientific Computing, King Faisal Specialist Hospital and Research Centre, Riyadh 11461, Saudi Arabia; DColakkaya@kfshrc.edu.sa; 6 Department of Pathology, Clinical Biochemistry Unit, College of Medicine, King Saud University, Riyadh 11451, Saudi Arabia; amujamammi@ksu.edu.sa (A.H.M.); esabi@ksu.edu.sa (E.M.S.); 7 Department of Genetics, King Faisal Specialist Hospital and Research Center, Riyadh 11211, Saudi Arabia; 8 Department of Biochemistry and Molecular Medicine, College of Medicine, Alfaisal University, Riyadh 11533, Saudi Arabia; 9 Department of Chemistry, College of Medicine, Memorial University of Newfoundland, St. John’s, NL A1B 3V6, Canada

**Keywords:** dexamethasone, glucocorticoids, metabolomics, mass spectrometry, rats, amino acids, side effects

## Abstract

Dexamethasone (Dex) is a synthetic glucocorticoid (GC) drug commonly used clinically for the treatment of several inflammatory and immune-mediated diseases. Despite its broad range of indications, the long-term use of Dex is known to be associated with specific abnormalities in several tissues and organs. In this study, the metabolomic effects on five different organs induced by the chronic administration of Dex in the Sprague–Dawley rat model were investigated using the chemical isotope labeling liquid chromatography-mass spectrometry (CIL LC-MS) platform, which targets the amine/phenol submetabolomes. Compared to controls, a prolonged intake of Dex resulted in significant perturbations in the levels of 492, 442, 300, 186, and 105 metabolites in the brain, skeletal muscle, liver, kidney, and heart tissues, respectively. The positively identified metabolites were mapped to diverse molecular pathways in different organs. In the brain, perturbations in protein biosynthesis, amino acid metabolism, and monoamine neurotransmitter synthesis were identified, while in the heart, pyrimidine metabolism and branched amino acid biosynthesis were the most significantly impaired pathways. In the kidney, several amino acid pathways were dysregulated, which reflected impairments in several biological functions, including gluconeogenesis and ureagenesis. Beta-alanine metabolism and uridine homeostasis were profoundly affected in liver tissues, whereas alterations of glutathione, arginine, glutamine, and nitrogen metabolism pointed to the modulation of muscle metabolism and disturbances in energy production and muscle mass in skeletal muscle. The differential expression of multiple dipeptides was most significant in the liver (down-regulated), brain (up-regulation), and kidney tissues, but not in the heart or skeletal muscle tissues. The identification of clinically relevant pathways provides holistic insights into the tissue molecular responses induced by Dex and understanding of the underlying mechanisms associated with their side effects. Our data suggest a potential role for glutathione supplementation and dipeptide modulators as novel therapeutic interventions to mitigate the side effects induced by Dex therapy.

## 1. Introduction

Glucocorticoids (GCs) are highly effective anti-inflammatory and immunosuppressant drugs that are widely used worldwide. One in five American adults were shown to have used corticosteroids during a three-year period [1], while 0.9% of adults in the UK have used oral corticosteroids at any given time point [2]. GCs exert their biological effects by binding with GC receptors (GCRs) and activating genomic and non-genomic pathways [3]. GCRs are members of the superfamily of nuclear receptors of transcription factors that are expressed in nearly every human tissue, thus regulating diverse physiological and metabolic processes [4].

Dexamethasone (Dex) is a synthetic GC used clinically to treat inflammatory and immune-mediated diseases, such as arthritis [5], allergic reactions [6], and asthma [7], and as part of various chemotherapy protocols [8]. While Dex is commonly used over a broad range of indications, long-term use is generally avoided as it can be associated with an increased risk of adverse events in different tissues and organs [9]. The administration of Dex at a high dose and/or over a long period results in clinically undesirable cardiovascular, endocrine, and metabolic side effects, such as atherosclerosis, hypertension, diabetes mellitus, and the redistribution of body fat. Additionally, complications of extended exposure to Dex seriously affect numerous organs, among which are bone (e.g., osteoporosis), muscle (e.g., myopathy), the kidney (e.g., adrenal insufficiency), and the liver (e.g., fatty liver) [3,10,11].

Dexamethasone side effects are associated with several metabolic abnormalities. Metabolomics is a rapidly growing analytical approach that traces changes in the levels of small biomolecules (i.e., sugars, amino acids, and nucleotides) in individual biological samples in response to external stimuli [12]. Pharmacometabolomic studies measure metabolic level changes in biological matrices during or after a drug intervention to predict and evaluate its metabolism. Therefore, they provide a deep understanding of the drug pharmacokinetic profile and its pharmacodynamic responses in metabolic pathways [13].

The LC-MS platform has been widely used in metabolomics analysis [14]. However, conventional LC-MS analysis has many issues, such as difficulty differentiating weak signals from background noise and inaccurate metabolite quantification caused by ion suppression. To solve these problems, the chemical isotope labeling (CIL) LC-MS method has been developed to offer a highly improved analytical performance for targeted or non-targeted metabolite analysis of various types of samples [15,16]. This chemical derivatization method can significantly enhance the electrospray ionization signal and improve the reversed-phase (RP) LC separation.

Metabolomics analysis has been used to investigate the toxicity of different drugs in the serum and liver of rats [17]. Previous studies have reported on the metabolic changes associated with GCs in rat urine [18] and kidney tissue [19], and investigated the effect of Dex on lung metabolism in broncho-alveolar lavage fluid in mice [20]. Despite the available literature that has investigated the mechanisms of GC side effects, the majority of reported studies have mainly focused on studying single metabolic pathways or measuring the metabolic changes in a single biological fluid or within a single organ at a time [21]. Dex induces metabolic changes in a sophisticated fashion and impacts numerous organs and metabolic processes within the body. Therefore, exploring the metabolite profile in different organs provides a holistic understanding of the diverse altered cellular mechanisms and sheds light on the biochemical changes taking place in the diseased tissues.

In our previous targeted metabolomics study [22], we reported changes in the level of specific serum metabolites in rats treated with Dex and highlighted the clinical and morphological changes detected in the soft-tissue mass and the variation in organ sizes. Recently, we also applied a proteomic approach for the identification of changes in the proteome of major organs in Sprague–Dawley (SD) rats after long-term Dex therapy. The results of the proteomic study revealed the alteration of key enzymes involved in several metabolic biochemical pathways, including amino acids and nucleotide metabolism [9]. Based on our previous work on exploring the effect of chronic Dex treatment using new state-of-art strategies and the fact that Dex side effects are associated with several metabolic abnormalities that seriously affect numerous organs, in this study, we aimed to apply the metabolomics approach using the chemical isotope labeling liquid chromatography-mass spectrometry (CIL LC-MS) platform to target the amine/phenol sub-metabolomes in five individual organs (brain, heart, kidney, liver, and skeletal muscle) in the Dex-treated SD rat model. The amine/phenol submetabolome includes several small biomolecules (i.e., amino acids and nucleotides) that have complex biological functions and are involved in central metabolism pathways. Therefore, the identification of significantly altered metabolites between the control and Dex-treated groups will aid in understanding the underlying mechanisms related to Dex-induced adverse effects and facilitating the development of more specific prevention strategies against Dex complications.

## 2. Results

Several phenotypic and clinical changes (i.e., elevation in blood glucose and triglyceride levels), distinct morphological alterations in the soft-tissue mass, and variation in the organ size (i.e., muscle atrophy, and decreased brain and heart sizes) were observed in the Dex-treated animals compared to the control group [9,22].

In this study, a total of 49 rat tissue samples were collected from five different body organs. Brain (5 Ctrl, 4 Dex), heart (5 Ctrl, 5 Dex), kidney (6 Ctrl, 5 Dex), liver (4 Ctrl, 4 Dex), and skeletal muscle (6 Ctrl, 5 Dex) tissues were analyzed using CIL LC-MS to identify the submetabolomic changes associated with prolonged treatment with Dex. The metabolic expression in tissue samples obtained from each organ was compared by orthogonal partial least squares-discriminant analysis (OPLS-DA) to visualize any grouping or clustering of the data that could be consistently related to the morphological changes. Additionally, to evaluate significantly up- or down-regulated metabolites due to Dex treatment, we analyzed the detected metabolites using the volcano plot, applying false discovery rate (FDR)-corrected *p*-values (y-axis) and fold change (FC) (x-axis) thresholds of 0.05 and 1.2/0.83, respectively.

A three-tier ID approach was used to identify metabolites, as summarized in Table 1 [23]. In tier 1, the chemical isotope labeling library (CIL library; amine/phenol channel) was used to positively identify detected metabolites based on their accurate mass and retention time matches with those of library standards. In tier 2, the linked identity library (LI Library) was used for the identification of the remaining peak pairs; it provides high-confidence putative identification results based on accurate mass and predicted retention time matches. In tier 3, putative identifications of metabolites were based on accurate mass matches, against the MyCompoundID (MCID) library (zero-reaction library), and their predicted metabolic products from one and two metabolic reactions (one-reaction library and two-reaction library, respectively).

### 2.1. Metabolite Identification and Submetabolomic Changes in Brain Tissue

Out of 1421 detected unique peak pairs, 1272 pairs (89.5%) were positively identified or putatively matched. A total of 104 peak pairs were positively identified in tier 1, whereas 45 peak pairs were putatively identified with high confidence in tier 2. In tier 3, 328, 572, and 223 peak pairs were matched in the zero-, one-, and two-reaction libraries, respectively.

**Figure 1:**
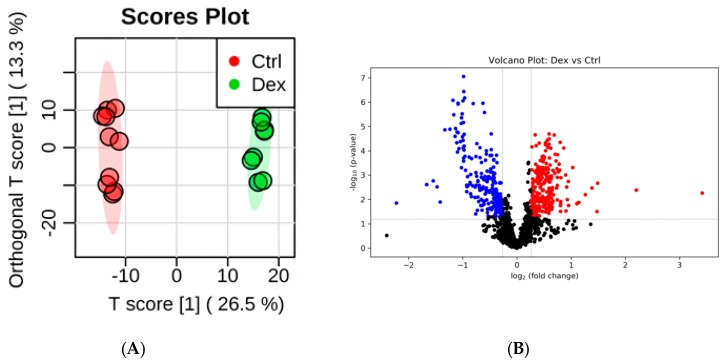

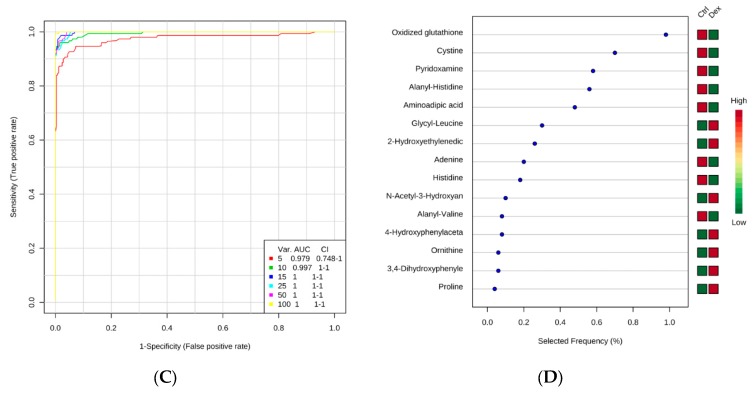
Rat brain tissue submetabolomic profile after prolonged treatment with dexamethasone (Dex). (**A**) Orthogonal partial least squares-discriminant analysis (OPLS-DA) score plot (*Q*^2^ = 0.892, *R*^2^ = 0.996) shows a clear separation between the healthy brain tissue (Ctrl, *n* = 5) and Dex-treated groups (*n* = 4). Tissue samples were run on LC-MS in duplicate. (**B**) Volcano plot shows the statistically significant altered metabolites (false discovery rate (FDR)-corrected *p*-value < 0.05, and fold change (FC) > 1.2 or < 0.83). The levels of 235 metabolites were down-regulated (blue) and 257 were up-regulated (red) in the Dex-treated rats. (**C**) The ROC curve was generated by the OPLS-DA model, with AUC values calculated from the combination of 5, 10, 15, 25, 50, and 100 metabolites. (**D**) Frequency plot shows 15 positively identified metabolites.

Prolonged treatment with Dex resulted in significant perturbations in the levels of several metabolites in brain tissues. Clear separation and grouping between brain tissue samples collected from Dex and control groups were demonstrated on the orthogonal partial least squares-discriminant analysis (OPLS-DA) score plot (Figure 1A), indicating differentially expressed metabolites between the two studied groups. The supervised OPLS-DA model yielded a satisfactory fitness of the model and predictive ability values (*R*^2^ = 0.996 and *Q*^2^= 0.892, respectively). Treating rats with Dex significantly perturbed the levels of 492 metabolites, of which 235 metabolites were down-regulated, while 257 were up-regulated, as shown in the volcano plot in Figure 1B.

Multivariate exploratory receiver operating characteristic (ROC) analysis was conducted using OPLS-DA as a classification and feature ranking method. The combination of the top 15 metabolites in the ROC curve gave the maximum confidence of differentiation and detection of Dex-treated rats from the control, with AUC = 1 (Figure 1C). The significant features of the positively identified metabolites are presented in Figure 1D.

The identity of the 37 positively identified metabolites that were significantly altered in Dex-treated animals are presented in Table 2. Among the identified altered metabolites in Dex-treated rats, cystine showed the most significant fold increase (2.21 compared to controls), whereas oxidized glutathione showed the most significant fold decrease (0.497 compared to controls), as presented in Table 2. The identity of all significantly altered metabolites is presented in Supplemental Table S1. In the brain, we detected 37 differentially expressed metabolites, including 16 (43%) dipeptides, of which 7 (50%) were proline-containing. However, one (6.2%) histidine-containing dipeptide (alanyl-histidine) was under-expressed.

### 2.2. Metabolite Identification and Submetabolomic Changes in Heart Tissue

The identification of detected masses, carried out using a three-tier ID approach, resulted in the positive and putative identification of 88.6% of the detected peaks (1378 peak pairs were identified out of the 1555 detected peak pairs). Among them, 114 were positively identified in tier 1; 39 were putatively identified with a high confidence in tier 2; and 325, 675, and 225 peak pairs were matched in the zero-, one-, and two-reaction libraries, respectively, in tier 3.

**Figure 2:**
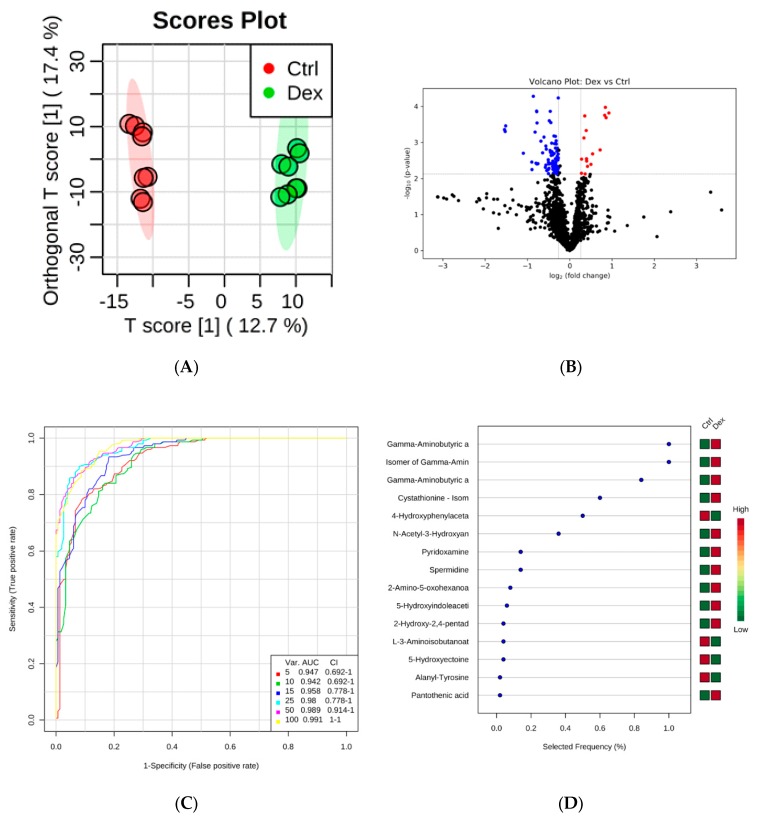
Rat heart tissue submetabolomic profile after prolonged treatment with dexamethasone (Dex). (**A**) OPLS-DA score plot (*Q*^2^ = 0.861, *R*^2^ = 0.987) shows a clear separation between the healthy group (Ctrl, *n* = 5) and Dex-treated groups (*n* = 5). Tissue samples were run on LC-MS in duplicate. (**B**) Volcano plot shows the statistically significant altered metabolites (FDR-corrected *p*-value < 0.05, and FC > 1.2 or < 0.83). The levels of 89 metabolites were down-regulated (blue) and 16 were up-regulated (Red) in Dex-treated rats compared to controls. (**C**) The ROC curve was generated by the OPLS-DA model, with AUC values calculated from the combination of 5, 10, 15, 25, 50, and 100 metabolites. (**D**) Frequency plot shows 15 positively identified metabolites.

Treating rats with Dex for an extended period leads to changes in the levels of multiple metabolites in heart tissues, as is evident in the OPLS-DA score plot (*R*^2^ = 0.987, *Q*^2^ = 0.861) in Figure 2A. The levels of 105 metabolites were significantly altered in Dex-treated animals, of which 89 metabolites were down-regulated and 16 were up-regulated (Figure 2B). Multivariate exploratory ROC analysis of the OPLS-DA model revealed AUC values ranging from 0.947 to 0.991 with the combination of the top 5–100 variables (Figure 2C). The significant features of the positively identified metabolites are shown in Figure 2D.

Table 3 lists the eight positively identified metabolites that were altered in the Dex-treated group. Except for uridine, all the remaining seven metabolites were down-regulated in Dex-treated rats. The ID information of all the significant differentially expressed metabolites is presented in Supplemental Table S2.

### 2.3. Metabolite Identification and Submetabolomic Changes in Kidney Tissue

Out of the 1538 unique peak pairs that were detected in the submetabolome of kidney tissue, 90.1% were positively identified or putatively matched. Using the CIL library, 136 peak pairs were positively identified, whereas 52 peak pairs were putatively identified with a high confidence in tier 2. In tier 3, 325, 631, and 242 peak pairs were matched in the zero-, one-, and two-reaction libraries, respectively.

**Figure 3:**
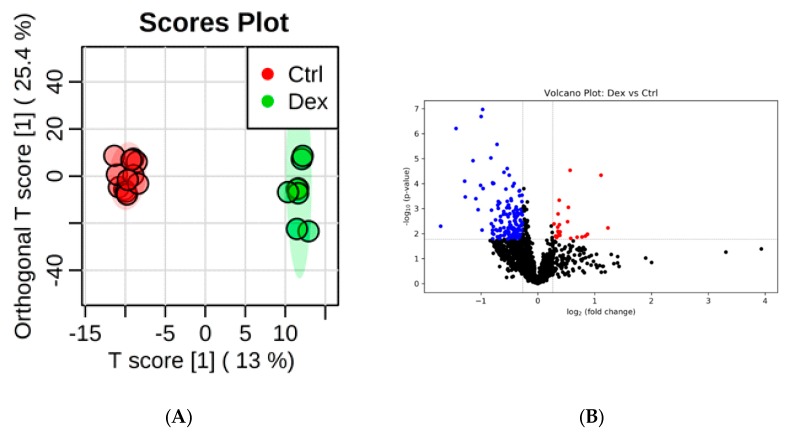

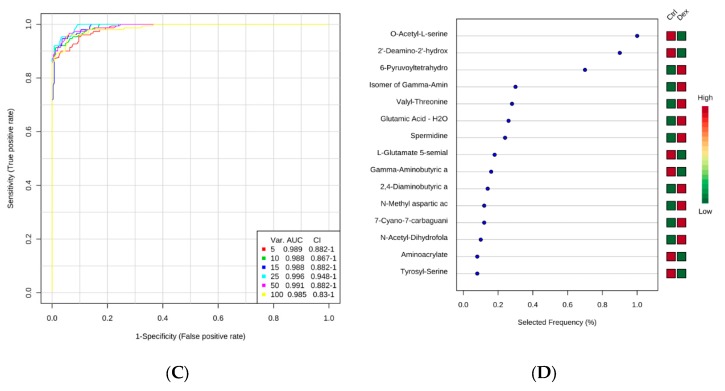
Rat kidney tissue submetabolomic profile after prolonged treatment with dexamethasone (Dex). (**A**) OPLS-DA score plot (*Q*^2^ = 0.886, *R*^2^ = 0.994) shows a clear separation between the healthy group (Ctrl, *n* = 6) and Dex-treated groups (*n* = 5). Tissue samples were run on LC-MS in duplicate. (**B**) Volcano plot shows the statistically significant altered metabolites filtered with q-value < 0.05, and FC > 1.2 or < 0.83. Out of 1538 detected metabolites, 162 were decreased (blue) and 24 increased (red) in Dex-treated rats compared to controls. (**C**) The ROC curve was generated by the OPLS-DA model, with AUC values calculated from the combination of 5, 10, 15, 25, 50, and 100 metabolites. (**D**) Frequency plot shows 15 positively identified metabolites.

As with the brain and heart tissues, dysregulation in the levels of multiple metabolites in kidney tissues was detected, as is evident in the apparent separation and grouping of the two studied groups in the OPLS-DA score plot, where *R*^2^ and *Q*^2^ values were 0.994 and 0.886, respectively (Figure 3A). The levels of 186 metabolites were significantly altered in kidney tissue in response to the prolonged intake of Dex. The levels of 24 and 162 metabolites were either increased or decreased, respectively, as shown in the volcano plot in Figure 3B. The ROC curve generated by the OPLS-DA model resulted in AUC values ranging from 0.985 to 0.996 (Figure 3C). A combination of the top 25 variables showed the maximum confidence of differentiation and detection of Dex-treated rats from the control, with AUC = 0.996 (Figure 3C). The significant features of the positively identified metabolites are presented in Figure 3D.

A total of 19 significantly altered metabolites were positively identified using the CIL Library (Table 3). The levels of glutamic acid, prolyl-glutamate, and 3-Aminoisobutanoic acid were increased in Dex-treated animals, whereas the levels of the remaining 16 metabolites were decreased (Table 4). The ID information of all significant metabolites is presented in Supplemental Table S3. The kidney metabolomic profile revealed six (32%) differentially expressed dipeptides, five (83%) of which were alanine-containing. β-alanine, the precursor for carnosine (hisitidine-containing) dipeptide, was down-regulated.

### 2.4. Metabolite Identification and Submetabolomic Changes in Liver Tissue

**Figure 4:**
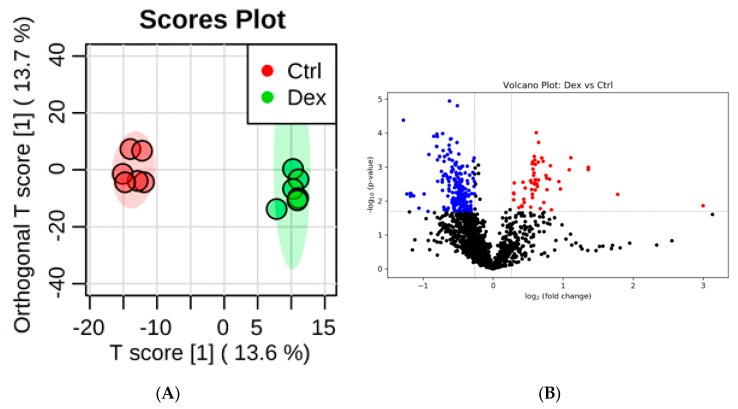

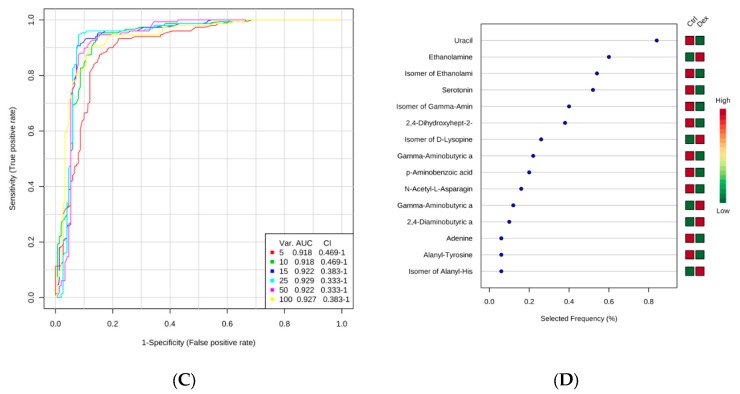
Rat liver tissue submetabolomic profile after prolonged treatment with dexamethasone (Dex). (**A**) OPLS-DA score plot (*Q*^2^ = 0.836, *R*^2^ = 0.991) shows a clear separation between the control group (Ctrl, *n* = 4) and Dex-treated groups (*n* = 4). Tissue samples were run on LC-MS in duplicate. (**B**) Volcano plot shows the statistically significant altered metabolites filtered with q-value < 0.05, and FC > 1.2 or < 0.83. The levels of 245 metabolites were down-regulated (blue) and 55 were up-regulated (red) in Dex-treated rats compared to controls. (**C**) The ROC curve was generated by the OPLS-DA model, with AUC values calculated from the combination of 5, 10, 15, 25, 50, and 100 metabolites. (**D**) Frequency plot shows 15 positively identified metabolites.

A total of 1586 unique peak pairs, corresponding to 87.1% of the total detected peaks, were positively identified or putatively matched using the three-tier ID approach (124, 45, and 360; 826; and 231 peak pairs were identified in tier 1; tier 2; and the zero-, one-, and two-reaction libraries in tier 3, respectively). Prolonged Dex therapy resulted in perturbations in the levels of several metabolites in liver tissues, as seen in the clear, complete separation and clustering of healthy and Dex-treated animal groups in the OPLS-DA score plot (*R*^2^ = 0.991, *Q*^2^ = 0.836) (Figure 4A). Volcano plot analysis (Figure 4B) revealed that of the 300 significantly differentially expressed metabolites, 55 were up-regulated and 245 were down-regulated, of which 253 were positively identified or putatively matched. The ROC curve generated by the OPLS-DA model revealed AUC values ranging from 0.918 to 0.929 (Figure 4C). The combination of the top 25 variables gave the ROC curve with the highest discrimination ability, with AUC = 0.929. The significant features of the positively identified metabolites are shown in Figure 4D.

The identity of the positively identified 20 metabolites that were significantly dysregulated in Dex-treated animals is presented in Table 4 (the identity of all significantly altered metabolites is presented in Supplemental Table S4). The levels of only five metabolites (out of 20) were increased, while the levels of the remaining metabolites were decreased, upon prolonged administration of Dex. Uracil and N-α-acetyllysine showed the highest increase and decrease in their levels, respectively, in Dex-treated animals compared to controls (Table 5). In the liver, nine dipeptides out of 20 (45%) differentially expressed metabolites were identified, including one histidine-containing dipeptide (seryl-histidine), which was down-regulated. The expression of histidine itself (a precursor of the dipeptide carnosine) was also down-regulated.

### 2.5. Metabolite Identification and Submetabolomic Changes in Skeletal Muscle Tissue

The three-tier ID approach followed for metabolite identification positively identified and putatively matched 87.8% of the detected peak pairs. The 1575 identified peaks included 123 peak pairs positively identified in tier 1; 49 peak pairs putatively identified with a high confidence in tier 2; and 356, 786, and 261 peak pairs matched in the zero-, one-, and two-reaction libraries, respectively, in tier 3.

**Figure 5:**
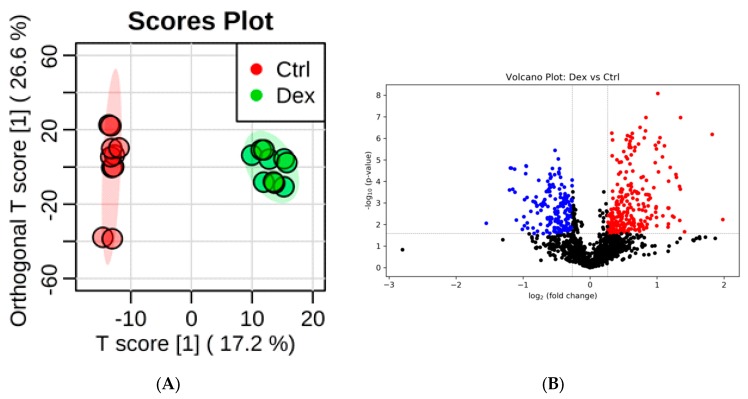

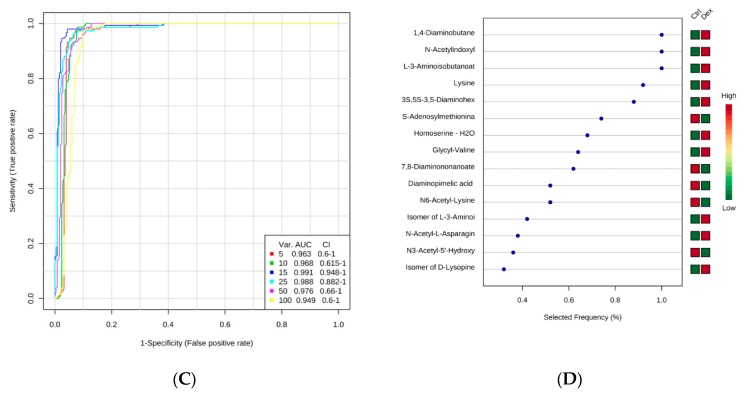
Rat skeletal muscle tissue submetabolomic profile after prolonged treatment with dexamethasone (Dex). (**A**) OPLS-DA score plot (*Q*^2^ = 0.928, *R*^2^ = 0.990) shows a clear separation between healthy muscle tissue (Ctrl, *n* = 6) and Dex-treated groups (*n* = 5). Tissue samples were run on LC-MS in duplicate. (**B**) Volcano plot shows the statistically significant altered metabolites (FDR-corrected *p*-value < 0.05, and FC > 1.2 or < 0.83). The levels of 268 metabolites (red) were increased and 174 were decreased (blue) in Dex-treated rats compared to controls. (**C**) The ROC curve was generated by the OPLS-DA model, with AUC values calculated from the combination of 5, 10, 15, 25, 50, and 100 metabolites. (**D**) Frequency plot shows 15 positively identified metabolites.

The OPLS-DA model (*R*^2^ = 0.990 and *Q*^2^ = 0.928) resulted in a clear separation between the two groups (Figure 5A), indicating that prolonged treatment with Dex induces significant changes in the levels of several metabolites in the skeletal muscle tissue. A total of 442 metabolites were significantly differentially expressed; 268 were up-regulated, and 174 were down-regulated, as shown in the volcano plot (Figure 5B). Of the altered metabolites, 374 were positively identified or putatively matched. The combination of the top 15 metabolites in the ROC curve analysis showed the maximum confidence of differentiation and detection of Dex-treated rats from the control, with AUC = 0.991 (Figure 5C). The significant features of the positively identified metabolites are shown in Figure 5D.

Table 6 represents the 13 positively identified metabolites that were significantly altered in Dex-treated rats. The levels of 1,4-diaminobutane showed the most significant increase in the Dex group, while the levels of oxidized glutathione exhibited the most significant decrease. All significantly perturbed identified metabolites are presented in Table S5 in the supplemental material.

## 3. Discussion

**Figure 6:**
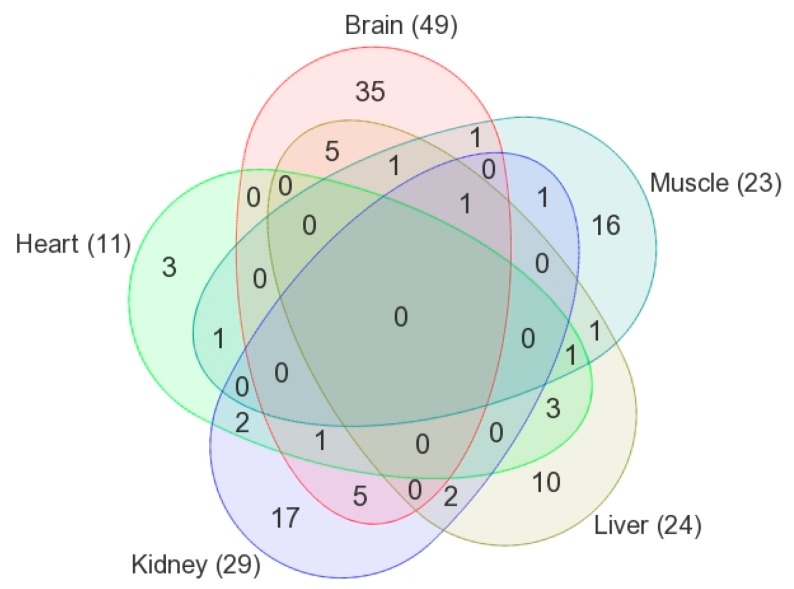
Venn diagram illustrating the number of shared and unique positively identified metabolites that are significantly altered in Dex-treated animals among different tissues (brain, heart, kidney, liver, and muscle).

A long-term intake of Dex is known to be associated with clinically undesirable side effects and can seriously affect numerous organs. In this study, we investigated the metabolic abnormalities linked to chronic exposure to Dex in five crucial organs using the isotope-labeled mass spectrometry-based submetabolomics approach. Our study revealed that a prolonged administration of Dex induced significant changes in metabolite levels in all studied organs. The OPLS-DA score plots constructed from tissue samples obtained from each organ showed a clear separation between control and Dex-treated groups for the brain, heart, kidney, liver, and skeletal muscle tissue samples. The common and specific metabolites among different tissues were highlighted in a Venn diagram for the statistically significant positively identified features, as shown in Figure 6. When compared to controls, a prolonged intake of Dex resulted in significant perturbations in metabolite levels in the brain and skeletal muscle tissues, with 492 (of which 49 were positively identified) and 442 (of which 23 were positively identified) metabolites being altered, respectively.

**Figure 7:**
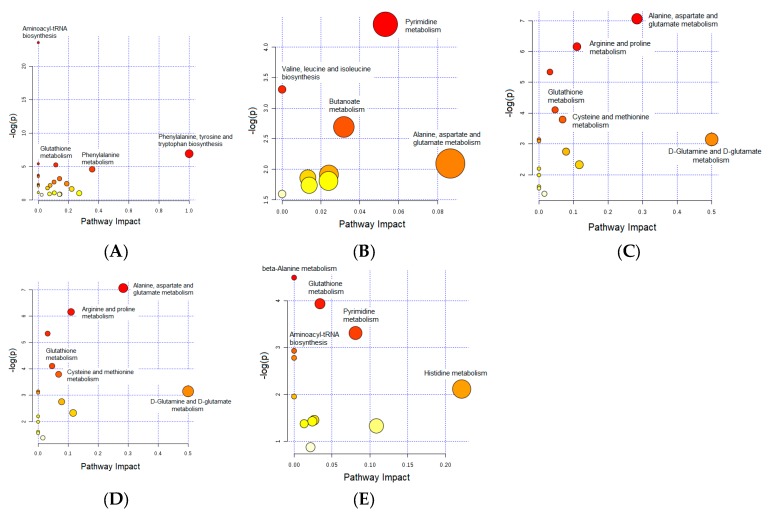
Pathway analysis of the positively identified and significantly altered metabolites in the (**A**) rat brain, (**B**) heart, (**C**) kidney, (**D**) liver, and (**E**) skeletal muscle tissues after prolonged treatment with dexamethasone. The size and color of each circle were based on the pathway impact value and *p*-value, respectively.

The effect was also pronounced, but to a lesser extent, in the liver and kidney tissues, with levels of 300 and 186 metabolites being changed, respectively. The least significant effect of Dex therapy occurred in the heart tissues, with only 105 metabolites being dysregulated. The levels of several metabolites were consistently changed in more than one organ. For instance, gamma-aminobutyric acid (GABA) was down-regulated in both heart and kidney tissues (Table 3 and Table 4), whereas uridine/uracil was up-regulated in both heart and liver tissues (Table 3 and Table 5). Even though none of the significantly altered metabolites could be positively identified in all organ tissues, oxidized glutathione was found to be significantly down-regulated in four of the studied organs, including the brain, kidney, liver, and skeletal muscle, reflecting significant perturbations in redox hemostasis. The majority of significantly altered metabolites were amino acids and dipeptides. Given the functional complexity of amino acids and their involvement in central metabolisms, pathway analysis performed using positively identified altered metabolites (Table 2, Table 3, Table 4, Table 5 and Table 6) revealed several metabolic pathways to be impaired in the organs of Dex-treated animals (Figure 7).

### 3.1. Important Pathways Altered in the Brain after Dex Treatment

In the brain, amino acyl-tRNA biosynthesis was the most significantly altered pathway (Figure 7A), pointing to widespread perturbations in protein biosynthesis and amino acid metabolism. The levels of several proteinogenic amino acids (i.e., histidine, proline, isoleucine, and valine) and dipeptides (i.e., glutamyl-leucine and valyl-serine), as shown in Table 2, were significantly increased in the Dex-treated group compared to controls, which confirms another study’s finding of the activation of proteolysis after GC administration [24]. Typically, the released amino acids play roles in energy production, gluconeogenesis, or the synthesis of acute-phase proteins [25]. However, in our study, the high levels of amino acids might also reflect the protein catabolism mechanism induced by chronic Dex treatment. Such substantial protein degradation could contribute to an irreversible loss of brain tissue, and might explain the decrease in brain weight reported in our proteomic study [9] and the increase in brain atrophy previously reported with chronic GC treatment [26]. 

Among the significantly affected pathways in the brain are phenylalanine, tyrosine, and tryptophan biosynthesis, where these three aromatic amino acids serve as precursors for the catecholamines (i.e., dopamine, epinephrine, substrate tyrosine, and to a lesser extent phenylalanine) and monoamine neurotransmitter serotonin (substrate tryptophan) [27]. Serotonin and catecholamine neurotransmitters are involved in pathways related to the pathophysiology of major depression and regulation of the hypothalamic-pituitary-adrenal (HPA) axis [28]. Therefore, disturbances in the levels of these amino acids point to alterations in brain excitability and psychological behavior. The initial step in catecholamine synthesis involves the hydroxylation of tyrosine to dihydroxyphenylalanine (DOPA) by the enzyme tyrosine hydroxylase. This step is considered to be the rate-limiting step and controls the synthesis of all catecholamines in this pathway [27]. GCs regulate catecholamine levels [29], and the action of tyrosine hydroxylase is increased by GCs [30]. Therefore, the increase in the brain concentration of tyrosine level detected herein is expected to affect the rates of synthesis/release of these neurotransmitters, leading to an increase in catecholamine levels and predictably altering brain functions [31]. Our results are consistent with those of Bordag et al. [32], who reported an increase in the plasma level of aromatic amino acids as a result of metabolome changes induced by Dex treatment in healthy male volunteers.

Disturbances in the redox status in the brain tissues as a consequence of the prolonged intake of Dex could be anticipated by the decrease in the levels of oxidized glutathione and pyridoxamine and the increase in the level of cystine. Advanced glycation endproducts (AGEs) have been linked to the development of degenerative conditions, including complications of diabetes mellitus and Alzheimer’s disease [33]. Pyridoxamine plays a crucial protective role against AGEs by inhibiting their formation through blocking oxidative degradation of the Amadori intermediate of the Maillard reaction and scavenging both reactive oxygen species (ROS) and the toxic products of lipid and glucose degradation [34]. Therefore, the lower level of pyridoxamine detected herein in the Dex-treated group might lead to the accumulation of AGEs and ROS and contribute to neurotoxicity [35]. Additionally, metabolites with a thiol group (cysteine/cystine and glutathione/oxidized glutathione redox couples) have an essential role in redox homeostasis [36]. Consequently, a higher level of the oxidized form of the amino acid cysteine, cystine, might reflect higher oxidation conditions in the brain.

### 3.2. Important Pathways Altered in the Heart after Dex Treatment

The number of metabolites affected by chronic Dex treatment in heart tissues was the lowest among the five studied tissues. However, several pathways were still altered, including pyrimidine metabolism; branched-chain amino acid (valine, leucine, and isoleucine) biosynthesis; butanoate metabolism; and alanine, aspartate, and glutamate metabolism, as shown in Figure 7B.

Pyrimidine nucleotides are precursors in the synthesis of DNA and RNA. Thymine resulting from the breakdown of DNA and RNA is broken into β-aminoisobutyrate, which is further split into compounds, eventually leading to the citric acid cycle [37]. Pyrimidine nucleotides can be synthesized from nucleosides recovered from RNA and DNA degradation in what is known as the salvage pathway. In the salvage pathway, uridine phosphorylase adds ribose 1-phosphate to the free base uracil, forming uridine. Through bonding with riboses and phosphates, uracil is required for the synthesis of many cellular enzymes, as an allosteric regulator and coenzyme for many important biochemical reactions. It is also involved in the biosynthesis of polysaccharides and the transportation of sugars containing aldehydes. [38]. The higher level of uridine and the decrease in the level of thymine detected herein (Table 3) indicated an alteration in pyrimidine biosynthesis/catabolism pathways, which will affect the metabolic and functional activities of cardiac cells.

The disturbances in butanoate and glutamate metabolism pathways and branched amino acid biosynthesis detected in this study all affected the level of non-proteinogenic amino acid gamma-aminobutyric acid (GABA). As a nitrogen donor, branched-chain amino acids contribute to the synthesis of excitatory glutamate and inhibitory GABA [39]. On the other hand, the initial step in butanoate metabolism involves the decarboxylation of glutamate to form GABA [40]. Although GABA is well-known as the major inhibitory neurotransmitter in the mammalian central nervous system, additional various biological activities in non-neuronal peripheral tissues and organs have been reported, including anti-hypertension, anti-diabetes, and anti-inflammation [41,42]. Additionally, reduced cardiac hypertrophy was observed in association with the oral administration of high GABA in spontaneously hypertensive rats [43]. The significantly lower level of GABA detected herein in the Dex-treated group might reflect a decrease in the level of circulating GABA and, therefore, might contribute to the higher blood pressure, increased insulin resistance, and cardiac hypertrophy associated with the prolonged administration of GCs [44].

### 3.3. Important Pathways Altered in the Kidney after Dex Treatment

The kidneys have a wide range of biological functions, including the urinary excretion of waste products, inter-organ exchange of amino acids, gluconeogenesis, and regulation of osmosis by maintaining acid-base equilibrium and electrolyte and fluid balances [45]. In our study, significant perturbations in several amino acid pathways in kidney tissues were identified with a chronic Dex intake (Figure 7C). This is in agreement with previous research that reported amino acid metabolism as one of the main categories altered in the kidney tissue of rats after the prolonged administration of prednisolone [19]. The altered pathways, particularly alanine, aspartate, and glutamate, and arginine and proline metabolism, reflected impairments in several biological functions of the kidney.

The urea cycle converts toxic ammonia to urea for excretion. Glutamine and arginine are components of the urea cycle [45], and, therefore, disturbances in the levels of these amino acids might indicate perturbation in ureagenesis. Additionally, arginine metabolism in the kidney is associated with arginine synthesis, arginine reabsorption, and creatine synthesis [46]. Creatine plays a significant role in the energy metabolism of tissues, whereas creatinine (formed via the non-enzymatic breakdown of creatine) is a well-established measure of renal functions. Hence, altered arginine metabolism might point to disturbances in renal functions. This is also supported by the lower level of GABA we detected in the Dex-treated group. In the kidney, GABA has a protective effect against toxin-induced damages, and its lower level will decrease the capacity of the kidney to tolerate toxins, resulting in tissue damage [42].

Glutamate, aspartate, asparagine, and alanine are derived from intermediates of central metabolism, mostly the citric acid cycle, also known as the Krebs cycle [47]. Besides its role in providing the precursor for the biosynthesis of specific amino acids, the Krebs cycle plays a starring role in the process of energy production of ATP [47,48]. The results of our study provide evidence that a chronic intake of Dex might be associated with disturbances in the Krebs cycle and, therefore, energy production and amino acid biosynthesis in the kidneys. Notably, our findings highlighted alterations in the level of several gluconeogenic amino acids (i.e., glutamate, aspartate, arginine, and alanine) reflecting perturbations in gluconeogenesis, which is in line with the work of Malkawi et al. [22], who reported disturbances in gluconeogenesis with prolonged Dex treatment in rats.

The present work sheds light on the effect of chronic Dex therapy on the regulation of osmosis, as noticed by the overexpression of glutamic acid in the Dex-treated group (Table 4). Glutamate is a precursor for arginine and glutamine, and the major intracellular nitrogen donor, and changes in its concentration accompany changes in osmolarity (acid-base balance) [49]. Our finding is consistent with the dysregulation in electrolyte and fluid balances that has been linked with GCs [50].

### 3.4. Important Pathways Altered in the Liver after Dex Treatment

Beta-alanine metabolism was the most significantly altered pathway in the liver of the Dex-treated group (Figure 7D). β-alanine is a non-essential amino acid that is involved in several biological functions. In the liver, β-alanine is degraded to eventually yield acetyl CoA, which is utilized by the Krebs cycle to produce energy [51]. Moreover, β-alanine has demonstrated a protective action against hypoxic liver injury [52] and is related to mitochondrial energy metabolism. Therefore, the impairment in β-alanine metabolism mainly suggested disturbances in energy production.

Among the other pathways altered in the liver is pyrimidine metabolism. Pyrimidine nucleotides, the information-carrying molecules of RNA and DNA, participate with their derivatives in cellular homeostasis and signaling and energy metabolism [53]. In the liver, uridine, the pyrimidine nucleoside, is in a continuous degradation and formation process that is essential to maintaining cellular homeostasis. Therefore, any disturbances of uridine homeostasis will have a direct impact on hepatic cellular functions. A previous study has reported a link between disturbances in uridine homeostasis and lipid accumulation in the liver and showed that the inhibition of uridine catabolism suppresses liver steatosis [53]. Evidence supports the formation of fatty liver and hepatic steatosis with the chronic administration of Dex [54]. Interestingly, one of the uridine catabolites, uracil, showed the highest fold increase among the identified altered metabolites herein, indicating that disturbances in uridine homeostasis shifted towards a uridine catabolism mechanism. Our finding can be linked to the reported fatty liver and hepatic steatosis induced with chronic Dex administration and showed that the uracil level might be a biomarker indicator of hepatic changes associated with a prolonged GC intake.

### 3.5. Important Pathways Altered in the Skeletal Muscle after Dex Treatment

Several pathways were significantly impaired in Dex-treated animals, including glutathione, arginine, proline, glutamine/glutamate, and nitrogen metabolism. The alteration of arginine and glutamine metabolism pointed to the modulation of muscle metabolism. Both amino acids are associated with muscle growth and protein synthesis. Arginine can protect myocytes from wasting during catabolic conditions [55] and is required for creatine synthesis to produce energy. Therefore, impairment in the arginine pathway reflected disturbances in energy production and muscle mass, which could be linked to muscle atrophy, a well-known side effect of prolonged GC intake [56]. On the other hand, glutamine is well-recognized for its involvement in various metabolic processes and is used as fuel by multiple tissues. Under stress conditions (i.e., catabolic state), glutamine is mobilized from the muscle tissue to provide energy for other tissues, resulting in the depletion of glutamine stores. In our study, the lower level of glutamine in muscle tissue of the Dex-treated group could be associated with the reported muscle atrophy induced by GCs [22]. This is a plausible conclusion given that glutamine could reverse GC-induced muscle atrophy by inhibiting myostatin, a negative regulator of skeletal muscle mass [57]. Our result is also in-line with the work of Salehian et al. [57], who showed that rats that received Dex plus glutamine displayed significantly less reduction in body and muscle weights compared with those treated with Dex alone.

### 3.6. Glutathione Metabolism: Commonly Altered Pathway in the Brain, Kidney, Liver, and Skeletal Muscle

One of the significantly altered pathways in the brain, kidney, liver, and muscle tissues in the Dex-treated group was glutathione metabolism (Figure 7). Glutathione (GSH) is a tripeptide composed of glutamate, cysteine, and glycine under the sequential effects of gamma-glutamate cysteine synthetase (GCLC), followed by glutathione synthetase (GSS). Recessively inherited mutations in either *GCLC* or *GSS* are thought to be rare and resulting symptoms range from asymptomatic hemolytic anemia to severe hemolytic anemia combined with metabolic acidosis, neurological deficits, and 5-oxoprolinuria. GSH is considered to be the central antioxidant defense agent against oxidant stress. GSH fulfills remarkable biological functions, including the detoxification of free radicals, scavenging ROS, nutrient metabolism, and regulation of body homeostasis [58]. It is converted to oxidized glutathione (GSSG) by glutathione peroxidase and can be recycled from the GSSG by glutathione reductase [59]. When the production of ROS exceeds the cellular antioxidant capacity, a condition of oxidative stress will occur, eventually leading to cell injury. In our study, only GSSG was identified with a high confidence as our experimental methods might have resulted in the oxidation of GSH. Typically, useful quantitation of both GSH and GSSG requires sensitive and specific techniques to make sure that the reduced state of the free thiol of glutathione will not be altered during sample preparation. Nevertheless, herein, GSSG was positively identified, and its level was significantly decreased in most organs, which highlighted the imbalance in the redox equilibrium of several tissues induced with prolonged Dex administration. This supports the association of chronic GC administration with both ROS and an imbalanced redox status that have been previously revealed in several tissues, including muscle and the nervous system [60]. The significance of the glutathione pathway was more pronounced in liver and muscle tissues, as indicated by pathway analysis (Figure 7D,E). The liver is one of the significant metabolic organs, and alteration of the redox homeostasis might negatively affect its metabolism and detoxification roles. In contrast, in the muscle, the redox impairment state might lead to mitochondrial dysfunction and muscle damage.

### 3.7. Dipeptide Profiling

In contrast to amino acids and proteins, l-α-dipeptides (dipeptides) have not been studied nearly as much, mainly because there have been ineffective production methods. The endogenous histidine-containing dipeptides carnosine (β-alanine-histidine) and anserine (β-alanine-l-methyl histidine), as well as the food additives aspartame (l-aspartyl-l-phenylalanine methyl ester) and Ala-Gln (l-alanyl-l-glutamine), are among the best-known dipeptides. Carnosine and anserine are stored in high concentrations in various tissues of multiple organs, without being incorporated into proteins. The biological functions of dipeptides are still emerging [61].

Using the taurine transporter, cells internalize β-alanine, which is the rate-limiting amino acid in the biosynthesis of carnosine [62], which is synthesized by the enzyme carnosine synthase (CARNS) and degraded by the hepatic enzyme carnosine dipeptidase 1 (CNDP1, carnosinase-1). Anserine and homocarnosine (g-aminobutyric acid-l-histidine) are also degraded by CNDP1. Hypercarnosinemia due to congenital carnosinase deficiency is rare and thought to be an isolated biochemical finding without a consistent clinical phenotype [63].

Carnosine and homocarnosine have potential neuroprotective and neurotransmitter functions in the brain [64]. Other protective functions of carnosine and anserine include pH buffering, the quenching of reactive oxygen species [65], and the degradation of advanced glycation (AGE) and lipoxidation (ALE) end-products [66]. Inhibition of the TGF-beta-mediated transcription of extracellular matrix proteins in both podocytes [67] and mesangial cells and the activation of podocytes PI3K/AKT and Nrf2 signaling pathways [68], among other mechanisms, result in the blocking of mesangial cell proliferation and podocyte apoptosis. In humans, a common leucine (CTG) repeat polymorphism in the signal peptide region of *CNDP1* (exon 2) has been associated with reduced carnosinase activity [69] and correlated with a significantly reduced risk for diabetic nephropathy and slower progression of chronic kidney disease due to glomerulonephritis [70]. In diabetic rodents, renoprotective (antiproteinuric and vasculoprotective) effects of carnosine have been demonstrated [71]. Since carnosine is short-lived in plasma, several alternative approaches aiming to increase the carnosine level have been pursued. Carnosinol, a new carnosine-mimetic compound [72]; carnostatin (chaperone-selective carnosinase inhibitor) [73]; and carnosine derivatives [74] have been developed.

In this study, for rats treated with Dex, disturbances in the metabolism of histidine and histidine-containing dipeptides in the kidney and liver were detected, but not in the heart or skeletal muscles. β-alanine (kidney), histidine, and seryl-histidine dipeptide (liver) were down-regulated. It is not clear why the expression of carnosine was not altered.

In response to Dex therapy, the dipeptide profiles in the rats’ various organs were distinctively different, both quantitatively and qualitatively (Table 2, Table 3, Table 4, Table 5 and Table 6). The brain, liver, and kidney were most active, while the heart and skeletal muscles showed no differential expression of dipeptides. Moreover, the up-regulation of proline-containing dipeptides appeared to be restricted to the brain. Specifically, both prolyl-glycine and prolyl-valine were up-regulated. Interestingly, in the protozoan Tetrahymena pyriformis, proline-glycine dipeptide exhibited the strongest chemosensory (chemotactic) response and positive hormonal imprinting (pre-treatment), while prolyl-valine had almost no effect on chemotaxis and induced negative imprinting. The imprinter effect of the dipeptides containing the same amino acids was dependent on the position, with positive imprinting only being induced with proline in the amino-terminal position [75]. It is not known if such a phenomenon also exists in animals. Therefore, it is not possible to predict the net effect of those two up-regulated proline-containing dipeptides in the brains of these rats.

Surprisingly, multiple alanine-containing dipeptides appear to be specifically differentially expressed in rat kidneys post-Dex therapy. Since we are unaware of any specific data in the literature about the biological functions of such dipeptides, it is hard to predict any secondary effect they may have on the kidney function. However, we noticed the down-regulation of β-alanine (which is essential for carnosine synthesis), which suggests the under-production of carnosine in the kidney. This prediction was not observed in our data.

### 3.8. Clinical Implications

Steroids are widely used in clinical practice, despite their potential significant side effects. Currently, there are 1602 and 569 human clinical trials that involve the use of Dex and prednisone, respectively (clinicaltrials.gov). Given the known side effects and emerging data on the diverse metabolomics abnormalities induced by Dex, we strongly recommend that global metabolomics studies become an integral tool in human clinical trials. Our metabolomics data suggest that glutathione and dipeptides are potentially relevant therapeutic biomarkers that could be clinically exploited to mitigate Dex-related side effects.

## 4. Materials and Methods

### 4.1. Ethical Considerations

Ethical approval for procedures and protocols for animal studies was given by the Animal Care and Use Committee (ACUC) at King Faisal Specialist Hospital and Research Center (KFSHRC) (approval number RAC2150016).

### 4.2. Experimental Design

Male Sprague–Dawley (SD) rats (age: 6 to 8 weeks, weight: 200–250 g) were housed in the animal facility of the Department of Comparative Medicine at KFSHRC (Riyadh, Saudi Arabia) under standard environmental conditions (Temperature: 20–24 °C, humidity: 45–50%, and 12 h/12 h light/dark cycle with ad libitum feeding and water intake) and standard protocol of housing. Details of the animal model used were reported previously [22]. Twenty rats were randomly separated into the Dex group (*n* = 10) and control group (*n* = 10). Rats in the Dex and control groups received a 2.5 mg/kg intramuscular injection of Dex or normal saline, respectively, twice a week, for 14 weeks, to induce most of the side effects associated with the prolonged administration of Dex, as suggested by Li et al. [76]. At the end of week 14, rats were sacrificed (5 rats/group), and five of their organs, namely, the brain, heart, kidney, liver, and muscle, were collected, snap-frozen in liquid nitrogen, and stored at −80 °C for subsequent metabolomics analysis. The remaining five animals in each group were used for radiological studies, as reported previously [22].

### 4.3. Chemicals and Reagents

Dexamethasone phosphate, analytical solvents, and other standard chemicals for metabolomics were obtained from Sigma–Aldrich (St. Louis, MO, USA). Lipid internal standard mix was purchased from Avanti Polar Lipids, Inc. (Alabaster, AL, USA). The LC-MS grade water, acetonitrile (ACN), methanol, and formic acid were purchased from Fisher Scientific (Ottawa, ON, Canada). The labeled compound ^13^C-Dansyl-chloride was synthesized in our lab, according to previously published procedures [77], and was available from the University of Alberta (http://mcid.chem.ualberta.ca).

### 4.4. Metabolomics

#### 4.4.1. Sample Preparation for Metabolite Profiling

**Figure 8:**
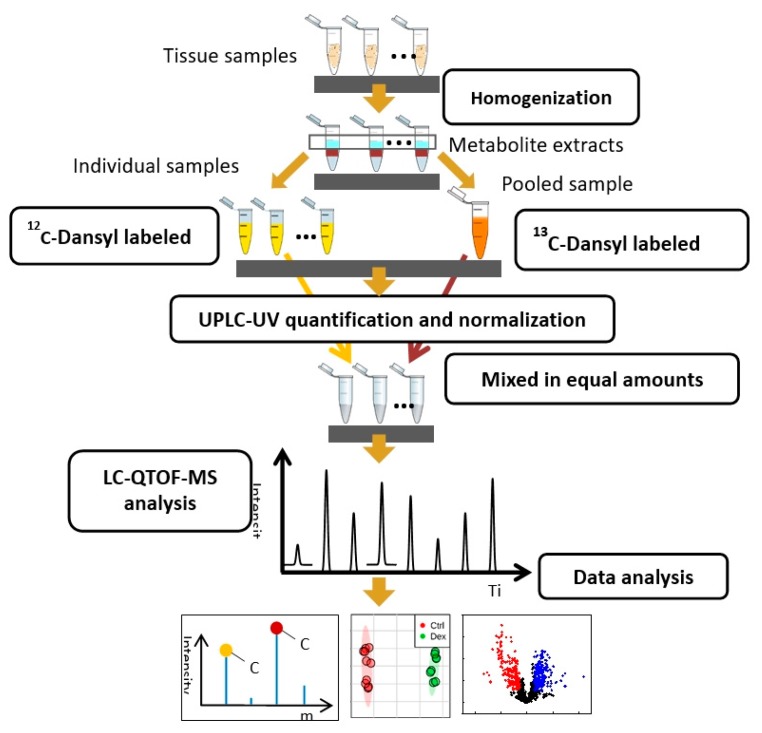
Workflow followed in the metabolomics profiling of rat tissues collected from five organs (different types of tissues were profiled separately). After 14 weeks of twice-weekly treatment of Sprague–Dawley rats with Dexamethasone (Dex), the amine/Phenol sub-metabolomes of freshly collected tissue samples from five major body organs were studied using chemical isotope labeling liquid chromatography-mass spectrometry. The metabolite profile was compared for the untreated control and Dex-treated groups for each studied organ.

Homogenized tissue lysates were prepared from rat brain (5 Ctrl, 4 Dex), heart (5 Ctrl, 5 Dex), kidney (6 Ctrl, 5 Dex), liver (4 Ctrl, 4 Dex), and muscle (6 Ctrl, 5 Dex) tissues, and were subjected to metabolomics analysis, as illustrated in Figure 8. When possible, more than one sample/tissue/animal was collected. For the extraction of metabolites, 0.5 µL of the lipid internal standard mix (used as a reference for relative quantification for a future lipidomics study) was added to each mg of the tissue, followed by the addition of 1.5 µL of ice-cold methanol and 0.42 µL of water per mg of tissue. The tissue was homogenized, and 2 µL of ice-cold dichloromethane and 1 µL of water, per mg of tissue, were then added. The homogenized tissue was vortexed, incubated at −20 °C for 15 min, and centrifuged at 12,000 rpm for 15 min at 4 °C. The aqueous supernatant layer was transferred to fresh tubes and used for metabolomics analysis. Following metabolite extraction, ^12^C-Dansyl labeling was carried out with the metabolite extracts of individual samples in duplicate, whereas ^13^C-Dansyl labeling was carried out with the metabolite extract of pooled samples [77]. The ^13^C-labeled pooled sample served as a reference for all ^12^C-labeled individual samples.

Samples were subjected to normalization and UPLC-UV quantification to determine the total concentration of dansyl-labeled metabolites based on a previously reported protocol [78]. The instrument for detection was a Waters ACQUITY UPLC system with a photodiode array (PDA) detector. A Phenomenex Kinetex reversed-phase C18 column (50 mm × 2.1 mm, 1.7 µm particle size, 100 Å pore size) was used to achieve a fast step-gradient. Mobile phase A was 0.1% (*v*/*v*) formic acid in 5% (*v*/*v*) ACN/water, and mobile phase B was 0.1% (*v*/*v*) formic acid in ACN. Starting at 0% B for 1 min, the gradient was increased to 95% B within 0.01 min and held until 2.5 min, to completely elute all labeled metabolites. Finally, the gradient was restored to 0% B in 0.5 min and held for another 3 min. The flow rate was 0.45 mL/min, and the total run time was 6 min. The peak area, which represents the total concentration of dansyl-labeled metabolites, was integrated using the Empower software (6.00.2154.003). According to the quantification results, before liquid chromatography-mass spectrometry (LC-MS) analysis by LC-QTOF-MS, each ^12^C labeled sample was mixed with equal molar amounts of ^13^C-labeled pooled samples. Meanwhile, the quality control (QC) sample was prepared by an equal amount of a ^12^C-labeled and ^13^C-labeled pooled sample.

#### 4.4.2. Metabolite Profiling Using LC-QTOF-MS

Metabolite profiling was carried out using a Thermo Fisher Scientific Dionex Ultimate 3000 UHPLC System (Sunnyvale, CA, USA) linked to a Bruker Maxis II quadrupole, time-of-flight (Q-TOF) mass spectrometer (Bruker, Billerica, UK). Metabolites were first separated using Eclipse plus C18 95 Å, 100 × 2.1 mm id, 1.8 μm column from Agilent (Santa Clara, CA, USA), and a mobile phase of 0.1% (*v*/*v*) formic acid in 5% (*v/v*) acetonitrile as solvent A and 0.1% (*v*/*v*) formic acid in acetonitrile as solvent B. The organic phase gradient from 0% to 99% of solvent B was applied as follows: t = 0 min, 20% B; t = 3.5 min, 35% B; t = 18 min, 65% B; t = 21 min, 99% B; t = 34 min, 99% B, with a flow rate of 0.18 mL/min. Separated metabolites were analyzed on Q-TOF-MS under positive mode under the following MS conditions: dry temperature, 230 °C; dry gas, 8 L/min; capillary voltage, 4500 V; nebulizer, 1.0 bar; endplate offset, 500 V; spectra rate, 1.0 Hz. Figure S1 represents an example to show the LC chromatogram obtained from a QC LC-MS injection. All labeled metabolites were identified as peak pairs on mass spectra, and the ratio of the average peak ratio value (^12^C-labeled individual sample vs. ^13^C-labeled pool) in one study group to that in the other study group was used for quantitative metabolomics analysis to obtain fold changes in the level of metabolites for the two studied conditions.

#### 4.4.3. Data Analysis and Informatics

Initially, all LC-MS raw data files were converted to CSV files by Bruker Daltonics Data Analysis 4.3 Software, UK, and peak pairs were extracted from CSV files by IsoMS. Mass pairs of adduct ions, such as Na^+^ and NH3^+^ adduct ions, were removed from the data set [79]. All data generated from multiple runs were aligned based on the peak’s accurate mass and retention time, and any missing values were filled by Zerofill software [80].

The processed raw data among the study groups for each organ were then uploaded to MetaboAnalyst version 3.0 (McGill University, Montreal, QC, Canada) [81] to analyze the expression profile of the metabolites in each organ. To ensure that all samples were normally distributed, the datasets were normalized to their total sample median for the specific organ. Data were log-transformed and Pareto-scaled to identify differentially expressed metabolites among the study groups and make individual features more comparable, respectively. For univariate analysis (volcano plot), the unpaired two-tailed Student’s *t*-test was used for a binary comparison of the different study groups. Metabolites visualized in a volcano plot with a false discovery rate (FDR)-corrected *p*-value of less than 0.05 and with a fold change greater than 1.2 (or less than 0.82) were considered significant. Multivariate analysis was carried out using orthogonal partial least squares-discriminant analysis (OPLS-DA) for modeling the differences between the studied groups using MetaboAnalyst version 3.0. The robustness of the created models was evaluated by monitoring the fitness of model (*R*^2^) and predictive ability (*Q*^2^) values. Models that yielded large *R*^2^ (close to 1) and *Q*^2^ (> 0.5) values were considered good models [82]. The receiver operating characteristic (ROC) curves were constructed using random forest method MetaboAnalyst software version 3.0 (McGill University, Montreal, QC, Canada) (http://www.metaboanalyst.ca) for global analysis. The Venn diagram approach was used to identify shared and unique metabolites among different tissues using PARTEK Genomics Suite (Partek Inc., St. Louis, MO, USA).

#### 4.4.4. Metabolite Identification

A three-tier ID approach was used to perform metabolite identification. In tier 1, peak pairs were searched against a chemical isotope-labeled metabolite library (CIL Library) based on an accurate mass and retention time. The CIL Library (amine/phenol channel) contains 712 experimental entries, including metabolites and dipeptides. In tier 2, the linked identity library (LI Library) was used for the identification of the remaining peak pairs. The LI Library includes over 2000 human endogenous metabolites from 68 metabolic pathways, providing high-confidence putative identification results based on accurate mass and predicted retention time matches. In tier 3, the remaining peak pairs were searched, based on accurate mass matches, against the MyCompoundID (MCID) library, which consists of 8021 known human endogenous metabolites (zero-reaction library) and their predicted metabolic products from one metabolic reaction (375,809 compounds; one-reaction library) and two metabolic reactions (10,583,901 compounds; two-reaction library) [83].

## 5. Conclusions

Systemic pharmacological actions of Dex are central for effective therapy and the management of adverse effects. Our study is the first to comprehensively investigate the metabolic side effects induced by the chronic administration of Dex in five vital body organs (brain, heart, kidney, liver, and skeletal muscle) in SD rats using a chemical isotope-labeled mass spectrometry-based metabolomics approach. In all five tissues, more than 1300 metabolites were detected, and more than 70% of those metabolites could be identified. Our results showed that long-term Dex therapy in rats resulted in significant metabolic changes in all tissues studied. Dex treatment triggered pronounced metabolic changes in the brain, skeletal muscle, and liver tissues, whereas it had less effect on the kidney and a minor impact on the heart tissues. The positively identified differentially expressed metabolites were mapped to diverse clinically relevant molecular pathways, among which glutathione metabolism, amino acid metabolism (notably glutamine, arginine, and aromatic amino acids), and pyrimidine metabolism showed a high significance in more than one tissue.

A direct comparison of our results with other metabolomics studies was difficult due to the limited number of studies that have investigated the effect of GCs on the levels of tissue metabolites, and the differences in the experimental conditions (treatments used and duration). However, the altered pathways discovered herein were associated with distinctive metabolic profiles, which correlate well with the reported Dex side effects. The identification of these pathways will provide better global insights into the molecular responses and associated mechanisms induced by Dex in damaged tissues. The significantly altered metabolites that were linked to the adverse effects of Dex therapy might serve as potential markers to monitor Dex-related adverse effects and develop prevention strategies.

## Figures and Tables

**Table 1:** Summary of the identified peaks and the differentially expressed metabolites in the five morphologically altered tissues after prolonged dexamethasone (Dex) administration.

Metabolic Identification	Brian	Heart	Kidney	Liver	Skeletal Muscle
Unique peak pairs detected (n)	1421	1555	1538	1821	1793
Positively identified or putatively matched (n) (%)	1272 (89.5%)	1378 (88.6%)	1386 (90.1%)	1586 (87.1%)	1575 (87.8%)
Tier 1 (n)	104	114	136	124	123
Tier 2 (n)	45	39	52	45	49
Tier 3 (Zero-reaction) (n)	328	325	325	360	356
Tier 3 (One-reaction) (n)	572	675	631	826	786
Tier 3 (Two-reaction) (n)	223	225	242	231	261
Differentially expressed metabolites					
Up-regulated (n)	257	16	24	55	268
Down-regulated (n)	235	89	162	245	174

n: number of metabolites.

**Table 2:** Positively identified significantly differentially expressed metabolites in the rat brain tissue after prolonged Dex treatment.

HMDB	Name	Neutral Mass (Da) *	Normalized RT (min) **	Fold Change	*p*-Value
HMDB00192	Cystine	240.0252	14.14	2.210	1.34 × 10^−2^
HMDB00177	Histidine	155.0697	18.24	1.727	6.28 × 10^−4^
HMDB00162	Proline	115.0636	10.19	1.611	2.24 × 10^−5^
HMDB00214	Ornithine	132.0911	16.38	1.554	8.83 × 10^−3^
HMDB00159	Phenylalanine	165.0792	12.76	1.541	7.83 × 10^−5^
HMDB0029016	Prolyl-Glutamate	244.1059	7.02	1.527	3.85 × 10^−4^
HMDB0029022	Prolyl-Lysine	243.1599	17.97	1.514	4.01 × 10^−3^
HMDB00696	Methionine	149.0512	10.91	1.488	7.29 × 10^−5^
HMDB00929	Tryptophan	204.09	11.49	1.475	3.71 × 10^−5^
HMDB0029030	Prolyl-Valine	214.1317	12.06	1.462	8.43 × 10^−3^
HMDB00172	Isoleucine	131.0949	13.02	1.442	5.62 × 10^−5^
HMDB00557	Alloisoleucine	131.0949	13.22	1.440	4.78 × 10^−5^
HMDB01645	Norleucine	131.0947	13.93	1.409	2.27 × 10^−4^
HMDB0028823	Glutamyl-Leucine	260.1386	9.92	1.404	2.03 × 10^−2^
HMDB0029026	Prolyl-Serine	202.0952	6.47	1.397	4.95 × 10^−4^
HMDB0011178	Prolyl-Glycine	172.0847	7.77	1.390	5.99 × 10^−4^
HMDB00158	Tyrosine	181.0754	22.65	1.366	4.90 × 10^−4^
HMDB0029015	Prolyl-Glutamine	243.1214	5.75	1.361	1.10 × 10^−2^
HMDB0029013	Prolyl-Aspartate	230.0902	6.85	1.355	1.56 × 10^−3^
HMDB0028928	Leucyl-Glutamate	260.1372	8.89	1.333	1.82 × 10^−3^
HMDB00883	Valine	117.0792	10.92	1.333	7.48 × 10^−4^
HMDB0029010	Prolyl-Alanine	186.1004	8.64	1.307	9.74 × 10^−4^
HMDB00300	Uracil	112.0275	11.28	1.299	1.28 × 10^−3^
HMDB0028736	Asparaginyl-Lysine	260.1438	12.10	1.285	4.11 × 10^−2^
HMDB0029136	Valyl-Serine	204.1107	6.28	1.282	3.62 × 10^−2^
HMDB00750	3-Hydroxymandelic acid	122.0371	21.21	1.278	8.05 × 10^−4^
HMDB00500	4-Hydroxybenzoic acid	138.0319	17.55	1.263	3.10 × 10^−3^
HMDB00182	Lysine	146.1068	17.50	1.259	7.52 × 10^−3^
HMDB0029101	Tyrosyl-Aspartate	296.1022	18.41	1.248	7.35 × 10^−3^
HMDB00123	Glycine	75.0322	6.61	1.235	3.83 × 10^−2^
HMDB00228	Phenol	94.042	23.15	1.212	1.33 × 10^−2^
HMDB28699	Alanyl-Tyrosine	252.1119	21.69	0.788	6.04 × 10^−3^
HMDB0000759	Glycyl-Leucine	188.1162	11.13	0.704	1.23 × 10^−3^
HMDB00510	Aminoadipic acid	161.0687	5.88	0.670	5.70 × 10^−3^
HMDB01431	Pyridoxamine	168.091	19.52	0.670	2.46 × 10^−4^
HMDB28689	Alanyl-Histidine	226.1068	17.44	0.607	8.03 × 10^−3^
HMDB03337	Oxidized glutathione	612.1526	7.99	0.497	3.02 × 10^−6^

* Neutral Mass (Da) is the neutral monoisotope mass of the metabolite (i.e., labeled mass—the mass of the labeling group). ** Normalized RT shows the corrected retention time of the peak pair with Universal RT Calibrant data.

**Table 3:** Positively identified significantly differentially expressed metabolites in the rat heart tissue after prolonged Dex treatment.

HMDB	Name	Neutral Mass (Da) *	Normalized RT (min) **	Fold Change	*p*-Value
HMDB00296	Uridine	244.0695	7.68	1.289	7.48× 10^−3^
HMDB00157	Hypoxanthine	136.0387	9.54	0.832	5.14× 10^−3^
HMDB00149	Ethanolamine	61.0528	6.11	0.829	7.16× 10^−3^
HMDB00755	Hydroxyphenyllactic acid	182.0579	14.27	0.786	5.65× 10^−3^
HMDB00167	Threonine	119.0584	5.80	0.704	3.02× 10^−3^
HMDB00763	5-Hydroxyindoleacetic acid	191.0584	14.80	0.584	3.95× 10^−3^
HMDB00262	Thymine	126.0431	13.10	0.538	3.50× 10^−3^
HMDB00112	Gamma-aminobutyric acid	103.0634	7.72	0.344	4.41× 10^−4^

* Neutral Mass (Da) is the neutral monoisotope mass of the metabolite (i.e., labeled mass—the mass of the labeling group). ** Normalized RT shows the corrected retention time of the peak pair with Universal RT Calibrant data.

**Table 4:** Positively identified significantly differentially expressed metabolites in the rat kidney tissue after prolonged Dex treatment.

HMDB	Name	Neutral Mass (Da) *	Normalized RT (min) **	Fold Change	*p*-Value
HMDB00148	Glutamic Acid	129.0428	9.33	1.484	2.94 × 10^−5^
HMDB0029016	Prolyl-Glutamate	244.1058	7.03	1.300	4.63 × 10^−4^
HMDB03911	3-Aminoisobutanoic acid	85.0524	16.11	1.258	1.42 × 10^−2^
HMDB0028922	Leucyl-Alanine	202.1319	10.47	0.826	1.26 × 10^−3^
HMDB0029010	Prolyl-Alanine	186.1005	8.64	0.813	1.07 × 10^−3^
HMDB00161	Beta-Alanine	89.0477	7.59	0.802	1.89 × 10^−4^
HMDB00161	Alanine	89.0475	7.92	0.801	3.04 × 10^−4^
HMDB29098	Tyrosyl-Alanine	252.1125	20.76	0.800	5.20 × 10^−3^
NA	Glycyl-Alanine	146.0691	6.20	0.779	1.41 × 10^−3^
HMDB00452	Alpha-aminobutyric acid	103.0634	9.16	0.778	1.13 × 10^−3^
HMDB00939	S-Adenosylhomocysteine	384.1222	10.87	0.766	1.66 × 10^−2^
HMDB01431	Pyridoxamine	168.0912	19.53	0.743	1.29 × 10^−3^
HMDB03337	Oxidized glutathione	612.1519	8.20	0.735	1.27 × 10^−2^
HMDB28680	Alanyl-Alanine	160.0846	6.34	0.722	4.04 × 10^−4^
HMDB00296	Uridine	244.0693	7.69	0.698	7.52 × 10^−4^
HMDB03464	4-Guanidinobutanoic acid	127.0737	10.74	0.642	3.55 × 10^−3^
HMDB02362	2,4-Diaminobutyric acid	118.0755	15.73	0.618	5.19 × 10^−4^
HMDB02393	N-Methyl aspartic acid	147.0532	7.27	0.609	2.71 × 10^−6^
HMDB00112	Gamma-aminobutyric acid	103.0633	7.72	0.576	7.33 × 10^−3^

* Neutral Mass (Da) is the neutral monoisotope mass of the metabolite (i.e., labeled mass—the mass of the labeling group). ** Normalized RT shows the corrected retention time of the peak pair with Universal RT Calibrant data.

**Table 5:** Positively identified significantly differentially expressed metabolites in the rat liver tissue after prolonged Dex treatment.

HMDB	Name	Neutral Mass (Da) *	Normalized RT (min) **	Fold Change	*p*-Value
HMDB00300	Uracil	112.0275	11.28	2.565	1.19 × 10^−3^
HMDB01414	1,4-Diaminobutane	88.1015	20.97	1.476	6.32 × 10^−3^
HMDB00149	Ethanolamine	61.0528	6.12	1.472	8.01 × 10^−4^
HMDB00500	4-Hydroxybenzoic acid	138.032	17.54	1.232	2.95 × 10^−3^
HMDB00089	Cytidine	243.0854	5.59	1.225	5.82 × 10^−3^
HMDB00177	Histidine	155.0698	18.23	0.822	6.65 × 10^−4^
HMDB0028823	Glutamyl-Leucine	260.1386	9.92	0.802	1.36 × 10^−2^
HMDB28848	Glycyl-Phenylalanine	222.1006	11.70	0.790	1.47 × 10^−2^
HMDB0029126	Valyl-Glutamate	246.1214	6.88	0.787	6.60 × 10^−3^
HMDB0029041	Seryl-Histidine	242.1019	15.36	0.761	1.42 × 10^−2^
HMDB0028907	Isoleucyl-Glycine	188.1153	8.84	0.753	1.62 × 10^−2^
HMDB0000759	Glycyl-Leucine	188.1162	11.11	0.751	6.20 × 10^−3^
HMDB00939	S-Adenosylhomocysteine	384.1228	10.87	0.736	1.30 × 10^−2^
HMDB00157	Hypoxanthine	136.0387	9.54	0.736	1.99 × 10^−2^
HMDB03337	Oxidized glutathione	612.1532	7.93	0.718	6.37 × 10^−3^
HMDB0029125	Valyl-Glutamine	245.1369	5.68	0.703	9.21 × 10^−3^
HMDB0028939	Leucyl-Threonine	232.1423	8.71	0.698	3.33 × 10^−3^
HMDB0029127	Valyl-Glycine	174.1004	7.32	0.688	8.67 × 10^−3^
HMDB00167	Threonine	119.0584	5.80	0.650	1.14 × 10^−5^
HMDB00446	N-Alpha-acetyllysine	188.116	6.85	0.576	1.27 × 10^−4^

* Neutral Mass (Da) is the neutral monoisotope mass of the metabolite (i.e., labeled mass—the mass of the labeling group). ** Normalized RT shows the corrected retention time of the peak pair with Universal RT Calibrant data.

**Table 6:** Positively identified significantly differentially expressed metabolites in the rat skeletal muscle tissue after prolonged Dex treatment.

HMDB	Name	Neutral Mass (Da) *	Normalized RT (min) **	Fold Change	*p*-Value
HMDB01414	1,4-Diaminobutane	88.1015	20.97	2.160	2.22 × 10^−6^
HMDB00099	Cystathionine	222.0686	13.69	1.867	5.93 × 10^−4^
HMDB01257	Spermidine	145.1594	10.34	1.478	1.18 × 10^−2^
HMDB00157	Hypoxanthine	136.0387	8.69	1.472	2.43 × 10^−3^
HMDB02390	3-Cresotinic acid	152.0477	16.80	1.369	2.98 × 10^−4^
HMDB00669	Ortho-Hydroxyphenylacetic acid	152.0479	16.49	1.368	3.03 × 10^−4^
HMDB00206	N6-Acetyl-Lysine	188.1161	5.66	1.288	1.03 × 10^−2^
HMDB00719	Homoserine	101.0478	9.01	1.268	6.07 × 10^−4^
HMDB00164	Methylamine	31.0423	9.73	0.831	1.58 × 10^−4^
HMDB00148	Glutamic Acid	129.0429	9.34	0.800	9.10 × 10^−3^
HMDB01370	Diaminopimelic acid	190.0967	12.97	0.689	2.05 × 10^−2^
HMDB00182	Lysine	146.1068	17.51	0.650	2.96 × 10^−4^
HMDB03337	Oxidized glutathione	612.1527	7.90	0.577	1.35 × 10^−3^

* Neutral Mass (Da) is the neutral monoisotope mass of the metabolite (i.e., labeled mass—the mass of the labeling group). ** Normalized RT shows the corrected retention time of the peak pair with Universal RT Calibrant data.

## Supplementary Materials

The following are available online at https://www.mdpi.com/2218-1989/10/2/42/s1, Table S1: List of peak pairs and identification information of metabolites significantly differentially expressed in brain tissue samples in the Dex-treated group compared to controls, Table S2: List of peak pairs and identification information of metabolites significantly changed in heart tissue samples in the Dex-treated group compared to controls, Table S3: List of peak pairs and identification information of metabolites significantly changed in kidney tissue samples in the Dex-treated group compared to controls, Table S4: List of peak pairs and identification information of metabolites significantly changed in liver tissue samples in the Dex-treated group compared to controls, Table S5: List of peak pairs and identification information of metabolites that were significantly changed in muscle tissue samples in Dex-treated group compared to controls, Figure S1: The LC chromatogram of a QC LC-MS injection.
