# Supplementary figures and images for: Dexamethasone-Induced Perturbations in Tissue Metabolomics Revealed by Chemical Isotope Labeling LC-MS Analysis
Source: Metabolites. 2020 Jan 21;10(2):42. doi: 10.3390/metabo10020042 (PMC7074358; doi:10.3390/metabo10020042)

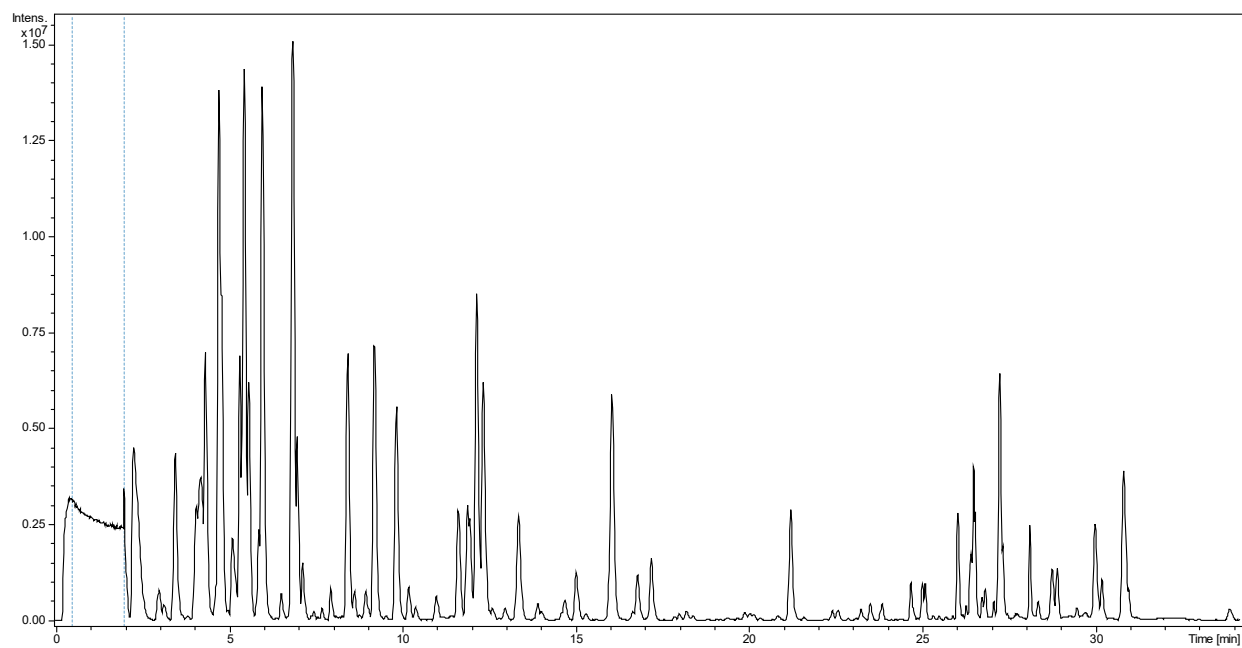

**Figure 1.** The LC chromatogram of a QC LC-MS injection.

Supplement: Supplementary file 1 [file metabolites-10-00042-s001.zip › Supplemental Figure S1.pdf]
